# Micromachining Microchannels on Cyclic Olefin Copolymer (COC) Substrates with the Taguchi Method

**DOI:** 10.3390/mi8090264

**Published:** 2017-08-29

**Authors:** Pin-Chuan Chen, Ren-Hao Zhang, Yingyot Aue-u-lan, Guo-En Chang

**Affiliations:** 1 Department of Mechanical Engineering, National Taiwan University of Science and Technology, Taipei 10607, Taiwan; m10403159@mail.ntust.edu.tw; 2 Materials and Production Engineering Program, The Sirindhorn International Thai-German Graduate School of Engineering (TGGS), King Mongkut’s University of Technology North Bangkok, Bangkok 10800, Thailand; yingyot.a.pe@tggs-bangkok.org; 3 Department of Mechanical Engineering, and Advanced Institute of Manufacturing with High-Tech Innovations (AIM-HI), National Chung Cheng University, Chia-Yi 62102, Taiwan; imegec@ccu.edu.tw

**Keywords:** cyclic olefin copolymer (COC), micromilling, rapid prototyping

## Abstract

Micromilling is a straightforward approach to the manufacture of polymer microfluidic devices for applications in chemistry and biology. This fabrication process reduces costs, provides a relatively simple user interface, and enables the fabrication of complex structures, which makes it ideal for the development of prototypes. In this study, we investigated the influence of micromilling parameters on the surface roughness of a cyclic olefin copolymer (COC) substrate. We then employed factor analysis to determine the optimal cutting conditions. The parameters used in all experiments were the spindle speed, the feed rate, and the depth of cut. Roughness was measured using a stylus profilometer. The lowest roughness was 0.173 μm at a spindle speed of 20,000 rpm, feed rate of 300 mm/min, and cut depth of 20 μm. Factor analysis revealed that the feed rate has the greatest impact on surface quality, whereas the depth of cut has the least impact.

## 1. Introduction

Microfluidics have been developing since the advent of micro gas chromatography in 1979 [1]. These developments have made it possible to conduct bio/chemical reactions on a small platform to lower reagent demand, accelerate reaction rates, minimize labor, reduce contamination, and enable integration with other functional components. These so-called micro total analysis systems (μTAS) are used in genetic analysis, clinical testing, drug discovery, food control, and environmental monitoring. Several low-cost fabrication methods have been reported to fabricate microfluidic platforms for bio-applications. For example, Pinto et al. [2] reported a method to fabricate a polydimethylsiloxane (PDMS) microfluidic chip, which used a cutting plotter to create patterns on adhesive papers as molds, followed by a PDMS casting and sealing process, to realize microfluidic chips for experiments. Liu et al. [3] reported a method for PDMS microfluidic chips, which used a laser to write patterns directly on photoresist as molds for a subsequent PDMS casting process. This article not only discussed this fabrication method, but also compared the cost, advantages, and drawbacks of different fabrication methods. Pinto et al. [4] used equipment for printed circuit board (PCB) industries instead of a cleanroom facility to create well-defined SU-8 microstructures with a minimum resolution of 10 μm and an aspect ratio of 20. Jang et al. [5] reported a method to fabricate glass microfluidic device for blood plasma separation, in which they used multiple replication processes to create glass microfluidic chips. Compared to other methods used for fabricating glass microfluidic chips, this method is relatively low-cost. To better understand the advantages and drawbacks of the reported low-cost fabrication methods for microfluidic chips, an article published by Faustino et al. [6] is highly recommended.

Micromachining is a time-efficient, low-cost approach to the manufacture of polymer microfluidic devices [7]. Unlike etching [8], lithography, electroplating, molding (Lithographie, Galvanoformung, Abformung, LIGA) [9,10], and PDMS casting [11], micromilling can be used on a wide range of materials to fabricate complex multi-level microstructures. Micromilling involves the mechanical removal of substrate material; therefore, the operating parameters, such as spindle speed, depth of cut, feed rate, and working environments, can greatly affect the surface quality of the resulting micromilled substrate. Growing interest in polymer microfluidic devices is driving the need for new methods used to prototype polymer microfluidic chips. Polymers are preferred for disposable microfluidic devices, due to their low cost, wide range of materials, and the maturity of manufacturing methods, such as injection molding.

Two methods have been proposed for the prototyping of polymer microfluidic devices using a micromilling machine: (1) milling a mold insert on metal substrates followed by hot embossing on the polymeric material, and (2) the direct milling of microchannels on polymer substrates. The productivity of the second approach tends to be somewhat lower; however, it is convenient for concept validation during the early stages of development. Direct milling involves only four steps to manufacture a device for testing: (1) design in computer-aided design (CAD), (2) conversion of the CAD file into G-code for the micromilling controller, (3) micromilling, and (4) bonding. The entire process generally takes less than 4 h to complete a ready-to-use chip for testing.

Computer numerical controlled (CNC) machines, such as lathes and mills, are commonly used in the manufacture of polymer microfluidic devices for chemical applications [12,13]. Researchers have sought to overcome the low accuracy, high surface roughness, and round corner features [14,15] by improving the manufacturing process [13] or using micromachining [16,17]. Many researchers have focused on micromilling a metal mold insert with a smooth surface quality for injection molding or hot embossing [18,19]. A critical concern of surface roughness in microfluidics is that a high surface roughness would influence the streamline in a small microchannel or affect the microfluidic device’s performance, especially in those cases which require surface force such as electrophoresis. For example, Hupert et al. compared the DNA separation efficiency on two types of microfluidic chip; one that was fabricated with the LIGA process and another that was fabricated with the micromilling process. They concluded that a smooth micromilled microchannel would not significantly affect the DNA separation performance [16].

Cyclic olefin copolymer (COC) is widely used in the manufacture of compact-disks and glasses. COC has been used in the manufacture of microfluidic devices for a variety of applications, such as polymerase chain reaction (PCR) [20], OLEDs [21], and biological microelectromechanical systems (BioMEMS) [22]. Spindle speed, feed rate, and depth of cut are the primary cutting parameters associated with micromilling; however, the final surface quality ultimately depends on the final cut. In this study, we examined the depth of cut at 10 μm, 15 μm, and 20 μm. We disregarded the effects of temperature and tool wear due to the softness of the polymer and the shallow cut depth. During the cutting process, we used compressed air to cool and clean the substrate. Our aim was to elucidate the effects of each cutting parameter on the surface roughness of a micromilled COC substrate. We then employed factor analysis to determine the optimal cutting parameters [23].

## 2. Experiment Design and Procedure

**Figure 1:**
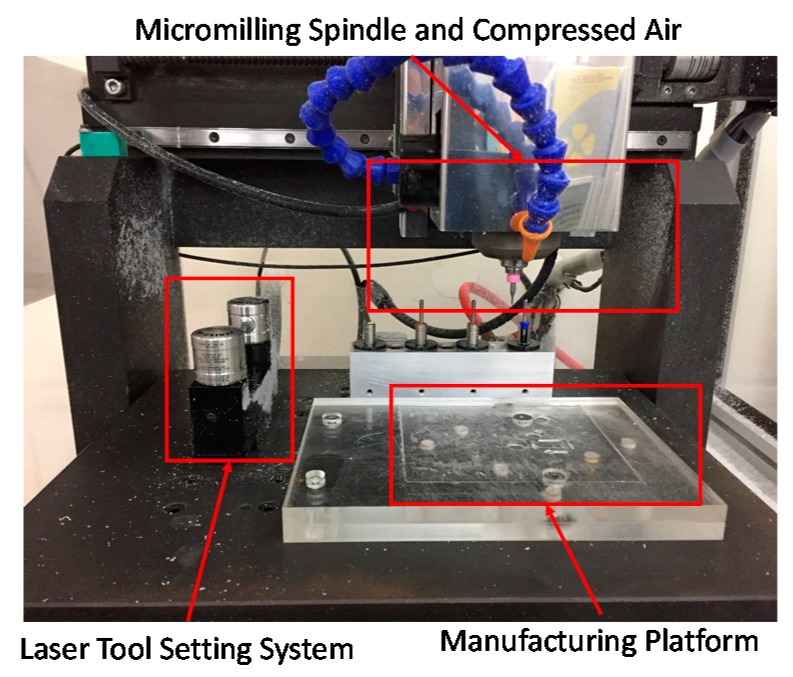
Micromilling system used in this study: five major components.

In this study, we used the micromilling machine in Figure 1. This device comprises five major components, including a micromilling spindle (E3000c, Nakanishi, Kanuma-Shi, Japan), a laser non-contact tool setting system (NC4, Renishaw, Wotton-under-Edge, UK), a numerical controller (M515i, LNC Technology Co. Ltd., Taichung, Taiwan), a compressed air/oil coolant system, and a bit holder for tool exchange. To minimize uncertainty, the COC substrates used in this study were self-manufactured by Professor Chang of National Chung Cheng University. The micromilling bit used in the experiment was a double-flute endmill with a diameter of 200 μm (Taiwan Microdrill Co. Ltd., Taipei, Taiwan).

**Figure 2:**
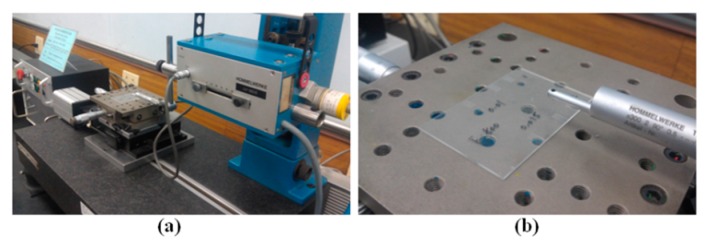
Stylus profilometer used in this study: (**a**) stylus profilometer; (**b**) measurement platform.

**Figure 3:**
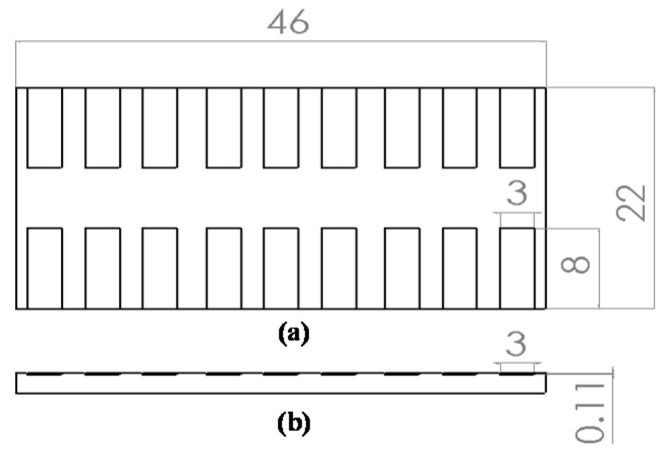
Dimensions of test sample in experiments: (**a**) top view; (**b**) side view.

The roughness of the micromilled COC substrates was measured at four points inside the reservoir using a stylus profilometer (Hommel Werke T400 & P2000 Pick-up TKL 300, Jenoptik, Jena, Germany) with a resolution of 0.01 μm (Figure 2). Figure 3 shows the 18 reservoirs micromilled on a single substrate using various cutting parameters. The reservoirs are 8 mm in length and 3 mm in width, and the depth is the sum of 0.1 mm plus the depth of the final cut (10 μm, 15 μm, or 20 μm). We designed a wide reservoir to avoid damage to the stylus from the wall of the reservoir during measurement.

As shown in Table 1, we adopted various parameter levels to elucidate the impact of spindle speed (N), depth of cut (DOC), and feed rate (F) on the micromilled surface. The three parameters studied herein are directly related to the surface roughness because they controlled the removal rate of material per flute. Table 2 lists the experiment conditions and the corresponding results based on the various levels of three factors. In all experiments, the step-over between each milling path was 20% of the diameter of the micromilling bit. Each reservoir was inspected using a measuring microscope to ensure that no significant scratching occurred on the milled surface as a result of a broken micromilling bit.

## 3. Results and Discussion

### 3.1. Experimental Result Analysis

**Figure 4:**
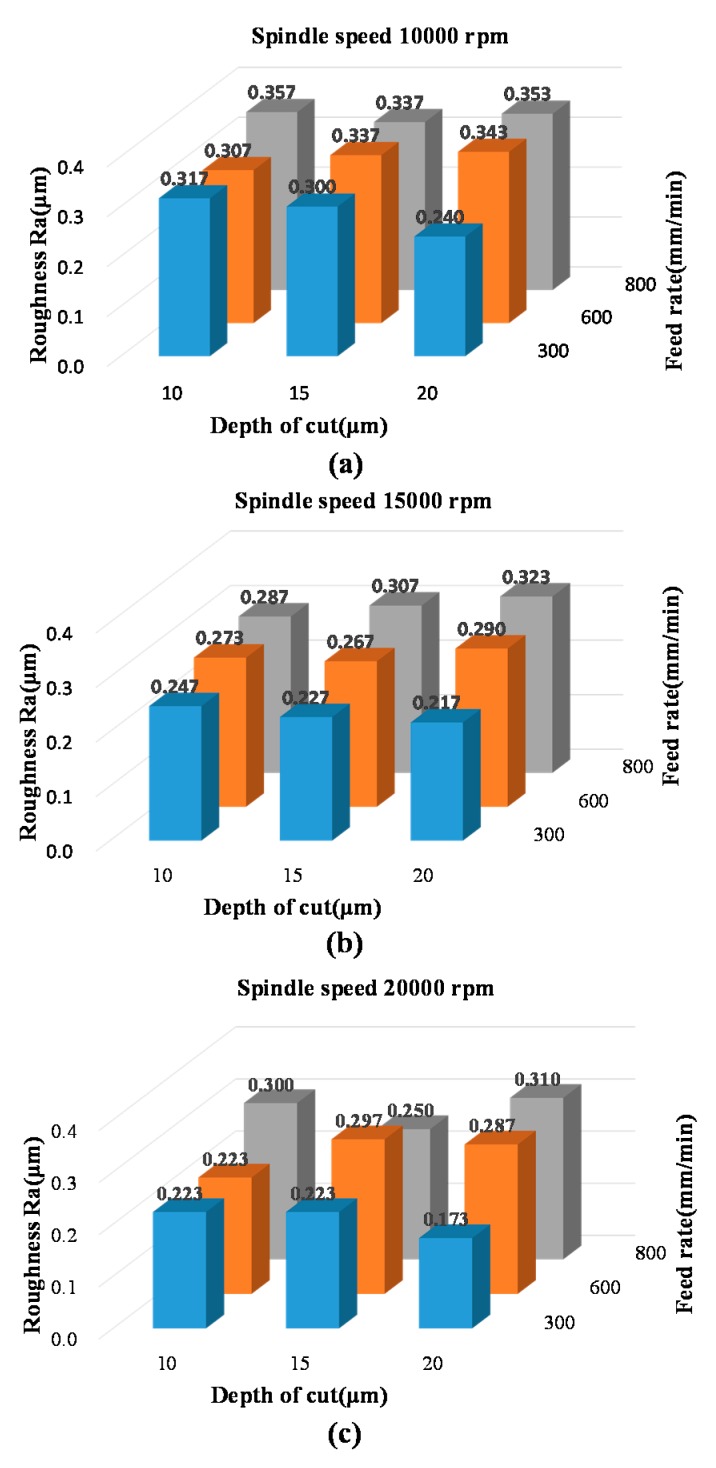
Roughness measurements obtained under various cutting conditions: Depth of cut (10 μm, 15 μm, 20 μm), feed rate (300 mm/min, 600 mm/min, 800 mm/min), and spindle speed (10,000 rpm, 15,000 rpm, 20,000 rpm): (**a**) measured surface roughness at spindle speed of 10,000 rpm; (**b**) measured surface roughness at spindle speed of 15,000 rpm; (**c**) measured surface roughness at spindle speed of 20,000 rpm.

Figure 4 shows the measured roughness values based on the cutting parameters listed in Table 2. All roughness values fell between 0.173 μm and 0.357 μm. Reducing the feed per flute (by increasing the spindle speed, reducing the feed rate, or reducing the depth of cut) was expected to reduce roughness; however, this did not prove to be true in the experimental results. This can be explained by the fact that the DOC in this experiment was on the same order as the edge radius of the micromilling bit (5 μm), which may have led to the incomplete removal of the COC substrate during the cutting process [2]. The results of the microscope analysis also revealed chips stuck to the micromilling bit, which may have affected the cutting process.

### 3.2. Factor Analysis

**Figure 5:**
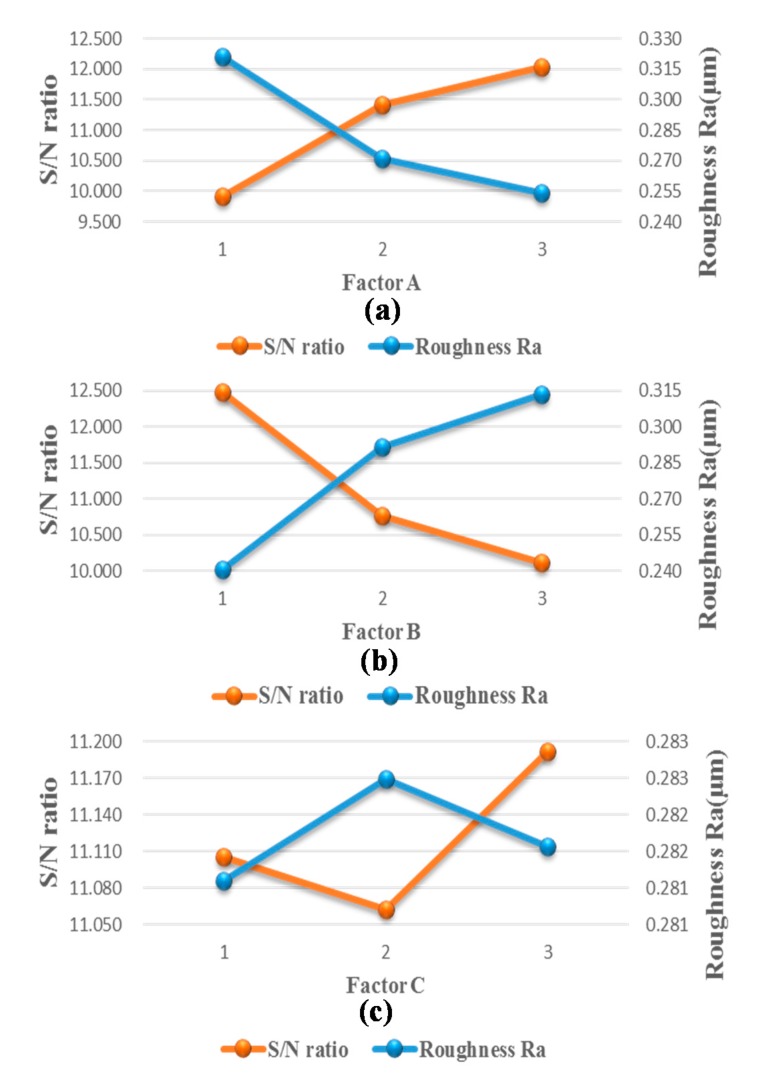
Calculated S/N ratio and measured roughness values associated with three major cutting parameters at three levels: (**a**) spindle speed, (**b**) feed rate, (**c**) depth of cut.

Factor analysis was used to identify the key cutting parameters involved in micromilling a microchannel directly on a COC substrate. Table 1 lists the three controlling factors with three levels each, which resulted in 27 combinations of cutting parameters. Table 2 lists the standard deviation (S) and signal-to-noise ratio (S/N) at four measurement points. Equation (1) [14] was used to calculate the signal-to-noise ratio (S/N), where *n* = 4 for four-time roughness measurements and *y_i_*^2^ is the sum of the four-time roughness measurements. Table 3 lists the average S/N ratio at each level to elucidate the impact of each factor (at different levels) on the surface roughness. Figure 5 shows the average S/N ratios in Table 3 as well as the average roughness (R_a_) values corresponding to the S/N ratios. These results show that the average roughness is inversely proportional to the average S/N ratio. In Table 3, the range of each factor is defined as the difference between the highest and the lowest average S/N ratios. A larger range indicates that the corresponding factor has a more pronounced effect on the surface quality of the micromilled COC substrate. Factor B presents the largest range (2.376), indicating that the feed rate has the greatest impact on surface quality. Factor C presents the smallest range (0.129), indicating that the DOC has the least impact on surface quality.

(1) $$S/N=-10\log\left[\frac{1}{n}\left(\sum {y_{i}}^{2}\right)\right]$$

Figure 5a–c show measured roughness in terms of feed rate and depth of cut based on three spindle speeds. Based on the criteria of low roughness and higher S/N ratio, Figure 5 can be used to identify the best cutting parameters to minimize the roughness of a micromilled COC substrate. The combination that resulted in the lowest roughness values (with a roughness of 0.173 μm and an S/N ratio of 15.219) was No 27 in the Table 2, which corresponds to a spindle speed of 20,000 rpm, a feed rate of 300 mm/min, and a depth of cut of 20 μm.

### 3.3. Confirmation Run

Experimental results revealed that the lowest roughness values were obtained from a spindle speed of 20,000 rpm, a feed rate of 300 mm/min, and a depth of cut of 20 μm. However, it is likely that these parameters are not directly applicable to other micromilling machines. Furthermore, several factors that are generally disregarded in conventional machining (substrate grain size and tool edge geometry), may actually play a dominant role in determining the surface quality when machining at the micro-scale [2]. To confirm our analysis, we micromilled 10 reservoirs on COC substrates using the optimal parameter settings identified in this study, the results of which are listed in Table 4. We employed the same measurement method to obtain values at four randomly selected locations in each reservoir for measurement using a stylus profilometer. The original roughness values obtained using the optimal cutting parameters was 0.173 μm; however, the average roughness in Table 4 was 0.183 μm, indicating a standard deviation of 0.02 μm.

## 4. Conclusions

Micromilling is a useful tool for the rapid prototyping of polymer microfluidic devices, particularly during the initial stages of development. This approach entails far lower costs and far less time than micromilling a metal mold insert followed by hot embossing a polymer microfluidic device. In this study, we sought to identify the optimal parameters for micromilling a COC substrate, with the aim of minimizing surface roughness. We focused on three parameters: spindle speed, feed rate, and depth of cut. Using 27 parameter combinations, measured roughness values fell between 0.173 μm and 0.357 μm. Factor analysis revealed that the feed rate has the greatest impact on surface roughness of a micromilled COC substrate, whereas the depth of cut has the least impact. The lowest roughness values in this study (0.173 μm) were obtained using a feed rate of 300 mm/min, a spindle speed of 20,000 rpm, and a depth of cut of 20 μm. The difference in roughness values between these two sets of cutting parameters falls within the resolution of the profilometer.

## Figures and Tables

**Table 1:** Three levels of three factors examined in this study: spindle speed, feed rate, and depth of cut.

Factor	Level 1	Level 2	Level 3
Spindle speed (N) (mm/min)	10,000	15,000	20,000
Feed rate (F) (mm/min)	300	600	800
Depth of cut (DOC) (μm)	10	15	20

**Table 2:** Experimental results based on the factors and levels listed in Table 1 (N: Spindle speed; F: Feed rate; DOC: Depth of cut; Average R_a_: Average roughness; S: Standard deviation; S/N ratio: Signal-to-noise ratio).

No.	N	F	DOC	Average R_a_	S	S/N Ratio
1	10,000	300	10	0.317	0.021	9.975
2	10,000	600	15	0.337	0.021	9.445
3	10,000	800	20	0.353	0.021	9.026
4	15,000	300	10	0.247	0.006	12.156
5	15,000	600	15	0.267	0.006	11.479
6	15,000	800	20	0.323	0.012	9.803
7	20,000	300	10	0.223	0.006	13.019
8	20,000	600	15	0.297	0.015	10.547
9	20,000	800	20	0.310	0.000	10.173
10	10,000	300	15	0.300	0.026	10.435
11	10,000	600	20	0.343	0.006	9.285
12	10,000	800	10	0.357	0.006	8.954
13	15,000	300	15	0.227	0.006	12.890
14	15,000	600	20	0.290	0.035	10.711
15	15,000	800	10	0.287	0.006	10.851
16	20,000	300	15	0.223	0.006	13.019
17	20,000	600	20	0.287	0.023	10.834
18	20,000	800	10	0.300	0.017	10.448
19	10,000	300	20	0.240	0.010	12.391
20	10,000	600	10	0.307	0.006	10.266
21	10,000	800	15	0.337	0.023	9.442
22	15,000	300	20	0.217	0.006	13.282
23	15,000	600	10	0.273	0.006	11.265
24	15,000	800	15	0.307	0.006	10.266
25	20,000	300	20	0.173	0.006	15.219
26	20,000	600	10	0.223	0.012	13.013
27	20,000	800	15	0.250	0.010	12.037

**Table 3:** Factor analysis results identifying key cutting parameters.

Levels and Response	A (Spindle Speed)	B (Feed Rate)	C (Depth of Cut)
Level 1	9.913	12.487	11.105
Level 2	11.412	10.760	11.062
Level 3	12.034	10.111	11.192
Range	2.121	2.376	0.129
Rank	2	1	3

**Table 4:** Measurement results of 10 samples with the best cutting parameters as a confirmation run.

No.	1	2	3	Average (R_a_) µm
1	0.14	0.14	0.15	0.143
2	0.18	0.19	0.18	0.183
3	0.20	0.20	0.20	0.200
4	0.15	0.14	0.15	0.147
5	0.18	0.20	0.19	0.190
6	0.21	0.20	0.20	0.203
7	0.17	0.17	0.18	0.173
8	0.19	0.19	0.18	0.187
9	0.19	0.20	0.20	0.197
10	0.20	0.22	0.21	0.210
